# Preparation and Characterization of Uniform and Controlled Silica Encapsulating on Lithium Yttrium Fluoride-Based Upconversion Nanoparticles

**DOI:** 10.3390/nano14080685

**Published:** 2024-04-16

**Authors:** Yahya A. Alzahrani, Abdulmalik M. Alessa, Mona K. Almosaind, Rahaf S. Alarifi, Abdulaziz Alromaeh, Masfer Alkahtani

**Affiliations:** 1 Future Energy Technologies Institute, King Abdulaziz City for Science and Technology (KACST), Riyadh 11442, Saudi Arabia; yalzhrani@kacst.edu.sa (Y.A.A.); mona.k.almosaind@gmail.com (M.K.A.); rahafalarifi11@gmail.com (R.S.A.); 2 Refining Technologies and Petrochemicals Institute, King Abdulaziz City for Science and Technology (KACST), Riyadh 11442, Saudi Arabia; aalessa@kacst.edu.sa; 3 Microelectronics and Semiconductors Institute, King Abdulaziz City for Science and Technology (KACST), Riyadh 11442, Saudi Arabia; eng.abdulaziz2@gmail.com

**Keywords:** lithium yttrium fluorides, upconversion nanoparticles, silica coating, recrystallization

## Abstract

In this work, we present an advancement in the encapsulation of lithium yttrium fluoride-based (YLiF_4_:Yb,Er) upconversion nanocrystals (UCNPs) with silica (SiO_2_) shells through a reverse microemulsion technique, achieving UCNPs@SiO_2_ core/shell structures. Key parameters of this approach were optimized to eliminate the occurrence of core-free silica particles and ensure a controlled silica shell thickness growth on the UCNPs. The optimal conditions for this method were using 6 mg of UCNPs, 1.5 mL of Igepal CO-520, 0.25 mL of ammonia, and 50 μL of tetraethyl orthosilicate (TEOS), resulting in a uniform silica shell around UCNPs with a thickness of 8 nm. The optical characteristics of the silica-encased UCNPs were examined, confirming the retention of their intrinsic upconversion luminescence (UC). Furthermore, we developed a reliable strategy to avoid the coencapsulation of multiple UCNPs within a single silica shell. This approach led to a tenfold increase in the UC luminescence of the annealed particles compared to their nonannealed counterparts, under identical silica shell thickness and excitation conditions. This significant improvement addresses a critical challenge and amplifies the applicability of the resulting UCNPs@SiO_2_ core/shell structures in various fields.

## 1. Introduction

Rare earths-doped upconversion nanocrystals (UCNPs) have attracted significant interest for their exceptional chemical stability, significant Stokes shifts, narrow emission bandwidths, and resistance to photobleaching and photo blinking, coupled with their minimal toxicity [1,2,3,4,5,6,7]. These optical and chemical properties make them an important class of optical materials for biomedical studies [8,9], optical refrigeration for remote cooling [10,11,12,13,14], phosphor for display applications (such as LEDs) [15,16], and luminescent thermometers [4,17,18]. To optimally harness the unique advantages of UCNPs for bio applications and other quantum technologies such as optical lasing and laser refrigeration, significant efforts have been put into choosing and improving the host materials [19].

The ideal host material for these nanocrystals must be transparent and capable of incorporating optically active lanthanide ions. Yttrium-based hosts are particularly suitable for this purpose due to the comparable size and oxidation state of Y^3+^ ions. Among these, lithium yttrium fluoride (YLiF_4_) is exceptional, meeting all these criteria while also being producible with superior optical and crystal quality [19,20]. Compared to NaYF_4_, the most popular and efficient host material for lanthanide-doped NCs, YLiF_4_ offers the advantage of a wide transparency range in the visible and near-infrared regions of the spectrum, which is essential for promising and efficient laser cooling [11,12,19,20,21]. Furthermore, lithium yttrium fluoride (YLiF_4_) upconversion nanoparticles (UCNPs) are distinguished by their unique square bipyramidal shape, attributed to the fact that the tetragonal crystal phase’s most stable crystal plane is the 011 plane. This results in eight surfaces with identical surface energies [19,20,22]. Such uniformity in surface energy allows for the easy and even application of various ligands or material functionalization onto the UCNPs, enhancing their suitability for biomedical uses [23,24].

To fully utilize the advantages of these promising YLF UCNPs in the above-mentioned applications, modifying their surface with a robust and controlled silica shell coating is required to enhance their colloidal and chemical robustness [25] and facilitate the attachment of ligands to the surface of YLF UCNPs [24,25,26], especially for bio applications. Furthermore, a silica coating on the surface of YLF UCNPS is highly effective for overgrowth of nanodiamond shells to produce a unique UCNPs@SiO_2_@diamond shell for applications such as laser refrigeration and optical trapping in quantum and biological technologies.

Toward this goal, the reverse microemulsion method is a well-known technique for adding a silica layer to small and hydrophobic NPs [26]. This approach depends on key parameters, including the NPs shape and concentration, an amphiphilic surfactant (Igepal CO-520), along with ammonia and tetraethyl orthosilicate (TEOS). The silica coating process starts by forming tiny soap-like bubbles that attach to the particles, allowing their surface to be modified and then coated with a silica layer through a specific and controlled chemical reaction [26].

In this study, we aim to synthesize high-quality lanthanide-doped YLiF_4_ UCNPs with an octahedral shape and to encapsulate them in precise and controlled silica shells. Also, we will introduce an effective method to avoid encapsulation of multiple UCNPs within a single silica shell with enhanced optical emission. We believe that this method will enhance the optical performance of the water-soluble, silica-coated UCNPs, making them very promising for future applications.

## 2. Materials and Methods

### 2.1. Materials

Ytterbium (III) chloride hexahydrate, yttrium (III) chloride hexahydrate, and erbium (III) chloride hexahydrate, which are classified as lanthanide elements or rare-earth chlorides, along with lithium hydroxide, ammonium fluoride, 28% concentrated ammonia, tetraethyl orthosilicate (TEOS), oleic acid, 1-octadecene, methanol, ethanol, cyclohexane, and deionized water, were sourced from Sigma-Aldrich, St. Louis, MO, USA. These chemicals were used as received, without undergoing any additional purification processes.

### 2.2. Synthesis of YLiF_4_:Yb,Er (18/2%) UCNPs Nanocrystals

To synthesize YLiF_4_:Yb,Er upconversion nanoparticles, start by dissolving LnCl_3_·6H_2_O (comprising 80% Y, 18% Yb, and 2% Er) in a mixture of oleic acid and 1-octadecene within a two-neck flask. Heat the mixture to 160 °C under argon for 40 min to achieve a clear solution, then cool to 60 °C. Following this, a prevortexed methanol solution containing LiOH·H_2_O and NH_4_F is injected into the flask. The mixture is maintained at 50 °C while stirring for nucleation, then heated to 160 °C to evaporate excess methanol and water. For crystallization, the temperature is raised to 300 °C and held for 1.5 h. Finally, the product is cooled, washed with ethanol, redispersed in cyclohexane, and stored at 4 °C for further use.

### 2.3. Preparation of YLiF_4_:Yb,Er@SiO_2_ UCNPs with Different UCNPs Contents

The production of YLiF_4_:Yb,Er@SiO_2_ upconversion nanoparticles (UCNPs) was conducted by adding reagents sequentially to a cyclohexane solution, held in a 50 mL round-bottomed flask at ambient temperature. The process began with mixing 30 mL of cyclohexane, 1.5 mL of Igepal CO-520, 0.25 mL of concentrated ammonia (28%), and 0.6 mL of a cyclohexane dispersion containing varying amounts (2.8 mg, 4 mg, 5 mg, 6 mg, and 7 mg) of YLiF_4_:Yb,Er UCNPs. It is important to note that the experiment was performed separately for each specified amount of UCNPs. Following the addition of the UCNPs, the solution underwent ultrasonic treatment for 10 min to become transparent. The next step involved coating the UCNPs with silica shells, achieved by adjusting the concentration of the tetraethyl orthosilicate (TEOS) that was added, which ranged from 10 to 50 µL. After stirring the mixture for 24 h at room temperature, the YLiF_4_:Yb,Er@SiO_2_ UCNPs were separated via centrifugation. The nanoparticles were then washed with ethanol, using centrifugation at 6000 rpm for 10 min, and redispersed in 4 mL of ethanol through ultrasonication for 10 min, resulting in a colloidal solution.

### 2.4. Preparation of YLiF_4_:Yb,Er@SiO_2_ UCNPs with Different Silica Shells

The preparation of YLiF_4_:Yb,Er@SiO_2_ UCNPs began by sequentially adding chemicals to a cyclohexane solution within a 50 mL round-bottom flask at room temperature. The process started with the addition of 30 mL of cyclohexane, 1.5 mL of Igepal CO-520, 0.25 mL of 28% concentrated ammonia, and 0.6 mL of a cyclohexane dispersion containing 7 mg of YLiF_4_:Yb,Er UCNPs. This mixture was then subjected to ultrasonic treatment for 10 min to form a clear solution. Next, to create YLiF_4_:Yb,Er@SiO_2_ UCNPs with various silica shell thicknesses, the concentrations of tetraethyl orthosilicate (TEOS) were varied between 10 and 50 µL, with each TEOS concentration tested in separate experiments. After adding TEOS, the mixture was stirred continuously for 24 h at room temperature. Finally, the YLiF_4_:Yb,Er@SiO_2_ UCNPs were separated using centrifugation, cleaned with ethanol by centrifuging at 6000 rpm for 10 min, and redispersed in 4 mL of ethanol through ultrasonication for another 10 min, producing a colloidal solution.

### 2.5. Recrystallization of YLiF_4_:Yb,Er UCNPs

In a dual-necked 50 mL round-bottom flask, a precise mixture of 8 mL of oleic acid (OA) and 12 mL of 1-octadecene (ODE) was prepared. Into this mixture, 2 mL of previously synthesized core upconverting nanoparticles (UCNPs) were added under an argon (Ar) atmosphere, deliberately omitting the addition of lithium (Li), yttrium (Y), and fluorine (F) ions. The solution was then heated to 110 °C to evaporate the chloroform, with continuous stirring throughout the process. After 40 min, the temperature was increased to a specified heat-treatment level of 240 °C and maintained for 1.5 h. Upon completing the heat treatment, the samples were allowed to cool down to room temperature gradually. The cooled UCNPs underwent a thorough washing process with ethanol three times to remove impurities, followed by redispersion in 2 mL of cyclohexane to obtain the final product.

### 2.6. Transmission Electron Microscope Imaging (TEM) of the Synthesized UCNPs@SiO_2_

TEM images of the synthesized UCNPs@SiO_2_ were captured using a high-resolution JEOL 2010 200 kV TEM (JEOL Ltd., Tokyo, Japan). To prepare for TEM imaging, the TEM grids were subjected to plasma treatment. This crucial step eliminated organic residues and dust and also enhanced the grids’ hydrophilicity, ensuring the sample solutions spread evenly. For the imaging process itself, we applied several drops of the synthesized UCNPs@SiO_2_ NP solutions onto these treated grids. Then, to ensure the samples were adequately prepared for high-quality imaging, they were dried in a vacuum environment.

### 2.7. Upconversion Emission Measurements of the Synthesized UCNPs@SiO_2_

We developed a specialized confocal laser scanning microscope tailored for the upconversion emission analysis of the synthesized UCNPs@SiO_2_ NPs. This optical microscope consists of a 4f imaging system, a high magnification microscope objective (100×, NA = 0.8, model number: LMPlanFL N, Olympus, Tokyo, Japan), Thorlabs GVS 212 Galvano scanners (Newton, NJ, USA), a continuous wave (CW) 532 nm green laser (CNI lasers, Changchun, China), and a custom-made spectrometer.

Prior to optical characterizations of the synthesized UCNPs@SiO_2_ NPs, we employed a drop-casting technique to evenly coat quartz substrates with the synthesized UCNPs and UCNPs@SiO_2_ NPs, ensuring a consistent and thin layer of the samples for uniform optical measurements. These substrates were then mounted on the confocal microscope’s x–y–z stage. The microscope’s x–y scanner facilitated the optical scanning of the samples with the green laser and then the capturing of the emitted optical emission spectra from the UCNPs@SiO_2_ NPs through the same objective lens. The spectra were analyzed using the home-made spectrometer equipped with a precise pinhole (Thorlabs, Newton, NJ, USA), an 1800-grooves/mm diffraction grating (Thorlabs, Newton, NJ, USA), achieving a high spectral resolution of 0.03 nm, a sensitive camera (Trius camera model SX-674, Starlight Xpress Ltd., Bottle Lane, UK), and a single photon counter (Hamamatsu photon counter model number H7155-21, Hamamatsu Photonics UK Limited, Welwyn Garden City, UK) for precise detection and quantification of emitted photons.

## 3. Results and Discussion

**Figure 1:**
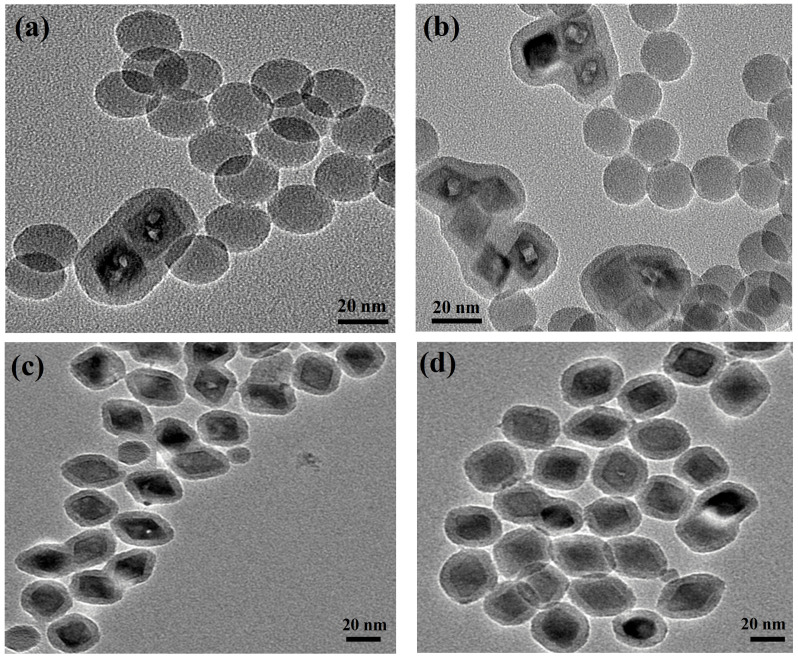
TEM images of UCNPs@SiO_2_ NPs with different content of UCNPs (**a**) 2.8 mg, (**b**) 4 mg, (**c**) 6 mg, and (**d**) 7 mg.

The YLiF_4_:Yb,Er UCNPs used in this study were synthesized following a hydrothermal synthesis procedure previously reported in [18,19,21] and detailed in Section 2. A silica coating of the synthesized YLiF_4_:Yb,Er UCNPs was performed using a reverse microemulsion method which was well reported and optimized for coating hydrophobic oleate-capped iron oxide (Fe_3_O_4_) nanoparticles [26]. This reverse microemulsion silica coating method is governed by the concentration of NPs, the surface polymer (Igebal CO-520), and the amount of ammonia and TEOS. For a successful silica coating of the synthesized YLiF_4_:Yb,Er UCNPs, first, we varied the content of the synthesized UCNPs from 2.8 mg to 7 mg to avoid core-free silica nanoparticles as summarized in Table 1. The silica coating procedure used in this study is experimentally detailed in Section 2. The amount of Igepal CO-520 and ammonia was carefully chosen to be 1.5 mL and 0.25 mL, respectively, which was found to strongly control the size and number of the aqueous domains during the silica formation. These values were optimized for a successful silica coating around NPs after many carful experiments reported in [26]. The experimental results revealed a significant presence of core-free silica particles, with only a few UCNPs successfully encapsulated in silica shells when employing 2.8 mg and 4 mg of the synthesized UCNPs, as depicted in Figure 1a,b. Addressing this issue, we increased the UCNP concentration to 6 mg, resulting in a remarkable reduction in core-free silica particles as evidenced in Figure 1c. By further adjusting the UCNP amount to 7 mg for the silica coating process, we achieved an optimal one-to-one encapsulation ratio of UCNPs within the silica, demonstrating a precise match between the number of UCNPs and the aqueous domains, as illustrated in Figure 1d.

**Figure 2:**
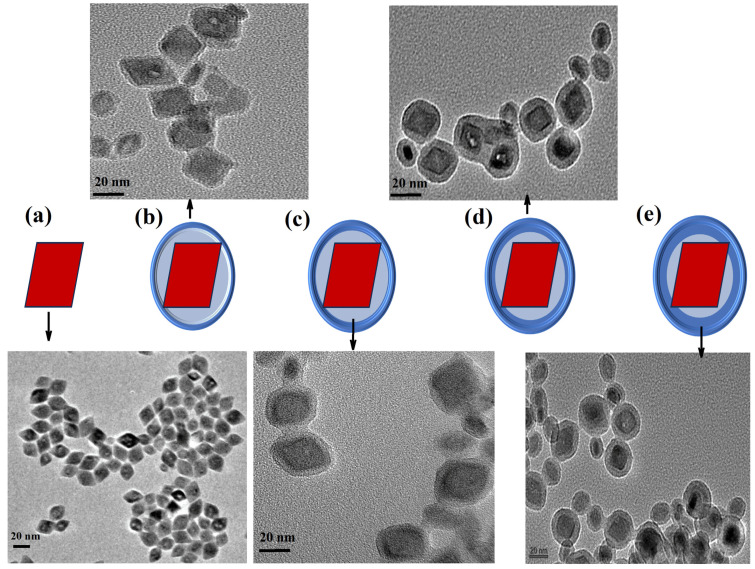
TEM images of UCNPs@SiO_2_ with different TEOS amounts. (**a**) UCNPs before silica coating. (**b**–**e**) UCNPs@SiO_2_ with different TEOS amounts ranging from 10 to 40 µL corresponding to silica coating thickness increases from 3.2 to 7.2 nm.

Prior to silica coating at different silica thicknesses, structural characterizations (morphology and size) of the synthesized YLiF_4_:Yb,Er UCNPs were performed using a transmission electron microscope (TEM). For this, diluted drops of UCNPs (10×) in cyclohexane were placed on a carbon-based TEM grid. Figure 2a shows well-dispersed octahedral nanoparticles with an average size of 20 nm. Upon optimizing the UCNP concentration, we achieved a uniform coating of the synthesized YLiF_4_:Yb,Er UCNPs with silica shells of varying thicknesses, as outlined in Table 2 and depicted in Figure 2b–e. The experimental methodology for this process is comprehensively described in Section 2. By maintaining a constant content of UCNPs at 7 mg, we systematically varied the tetraethyl orthosilicate (TEOS) volume from 10 μL to 50 μL. This adjustment, while keeping other parameters consistent as detailed in Table 2, allowed us to precisely control the silica shell thickness, which ranged from 3.2 nm to 8.1 nm. Notably, this method effectively prevented the formation of core-free silica particles, ensuring each UCNP was encapsulated within its own silica shell.

**Figure 3:**
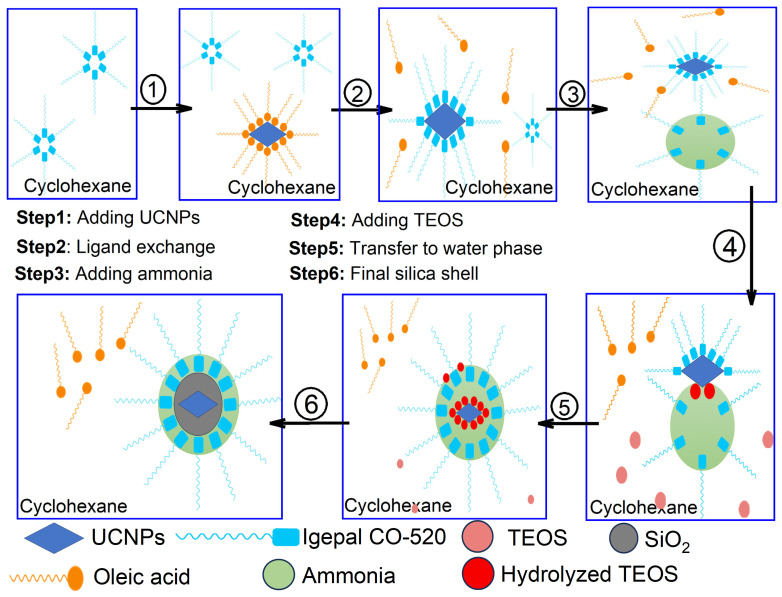
An illustration of the silica coating mechanism of silica shells around UCNPs.

To better understand the silica shell formation on the synthesized UCNPs via the reverse microemulsion process, an illustration schematic was put together as shown in Figure 3. At the first step, Igepal CO-520, a type of detergent, naturally groups together in the cyclohexane solution to form micelles due to its hydrophilic groups. These micelles form because Igepal CO-520 molecules have both water-loving and water-repelling parts. In the oil-like environment of cyclohexane, the water-repelling parts stick out, driving the molecules to cluster together. When oleate-capped UCNPs are introduced into this mix in step number two, an interesting ligand exchange takes place. Some of the oleic acid on the nanoparticles’ surface is replaced by Igepal CO-520, partially integrating the nanoparticles into the micelles. The addition of ammonia is the third and critical step, which causes the Igepal CO-520 micelles to absorb it and grow larger [25,26].

The Igepal CO-520 micelles will then be filled with ammonia upon its addition, and the micelle size is enlarged and forms a reverse microemulsion system. Subsequently in the fourth step, the added TEOS will hydrolyze at the oil/water interface and perform the ligand exchange with the Igepal CO-520 chemically absorbed on the UCNPs surface and will then transfer the UCNPs to the water phase. Finally in the last two steps, the hydrolyzed TEOS on the surface of the UCNPs undergoes a condensation process and forms silica shells.

The reverse microemulsion method is particularly advantageous for the synthesis of UCNPs@SiO_2_ core-shells due to its exceptional control over shell thickness and uniformity. This method allows precise manipulation of silica deposition on UCNPs’ cores by controlling the water-to-surfactant ratio and other reaction parameters within the microemulsion droplets. Unlike other synthesis techniques such as the Stöber process, which may produce larger and less uniform silica particles, reverse microemulsion facilitates the production of highly uniform silica shells at lower temperatures, preserving the quality of the sensitive cores. However, the technique has its drawbacks, such as the complex removal of surfactants from the final product, which can affect the purity and functionality of the silica shells. Moreover, scaling up this method for industrial production remains challenging due to the delicate balance of emulsion stability and the high cost of the surfactants and organic solvents used [26,27].

Comparatively, methods like sol–gel and hydrothermal are simpler and more cost-effective for producing silica particles at a high temperature, resulting in a lack of the fine control over shell morphology provided with reverse microemulsion [28].

**Figure 4:**
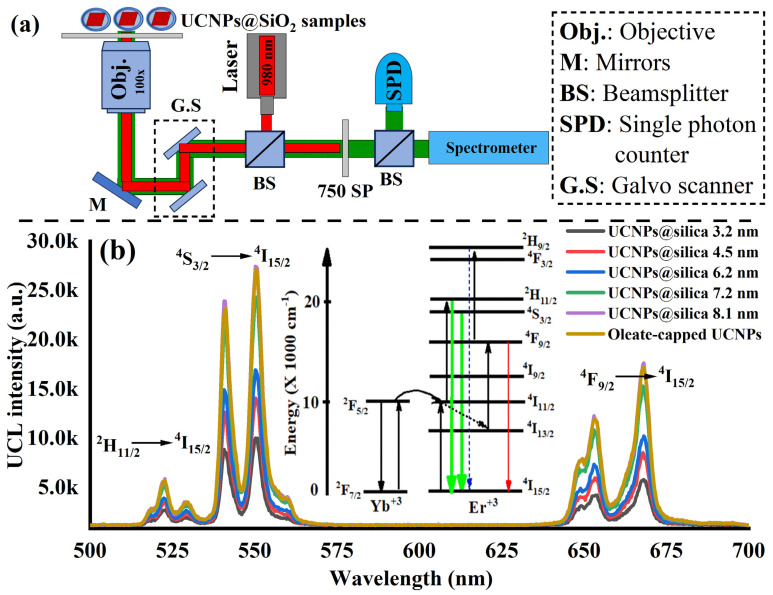
(**a**) An illustration of the custom-made optical microscope used for optical characterizations of UCNPs@SiO_2_. The optical setup consists of an imaging system with 100× objective, 980 nm laser directed into the samples with a beamsplitter (BS) and a custom-made spectrometer to analyze the recorded optical spectrum from the examined samples. The recorded emission spectra were filtered from the excitation laser with a 750 nm short pass filter (SP filter). (**b**) Optical emission spectra of the synthesized UCNPs coated with and without silica shells. It can be seen in this figure how the silica coating shell thicknesses helps UCNPs retain the good UC emission when dispersed in water to be more compatible for practical applications. (**b**, **inset**) An illustration shows the subsequent upconversion process in rare-earth ion-doped YLiF_4_ host crystals as they absorb near-infrared photons and emits visible photons.

Next, optical characterization of the synthesized UCNPs before and after silica coating was performed. For this purpose, a confocal scanning laser microscope was built and equipped with a near-infrared (NIR) laser (980 nm) and a custom-built spectrometer that covers a wide range of the spectrum from the visible (VIS) to NIR as illustrated in Figure 4a. The recorded emission spectra were filtered from the NIR excitation laser with a 750 nm short pass filter (SP filter). The optical microscope parts are detailed in Section 2. For optical testing, 1 µL of each sample (synthesized UCNPs and UCNPs@silica shells at different thicknesses) was spin-coated on a quartz slide to make a thin layer of the sample to avoid agglomeration during drying. The prepared substrates of the UCNP samples were then placed on the confocal microscope. Figure 4b shows the UC photoluminescence of the synthesized UCNPs before and after silica coating with two strong green emissions peaked at 520 nm and 550 nm corresponding to UC optical emission from both ^2^H_11/2_ and ^4^S_3/2_ excited states to the ^4^I_15/2_ ground state. In addition, there was a weak red emission that peaked at 650 nm, corresponding to the UC optical transition from the ^4^F_9/2_ excited states to the ^4^I_15/2_ ground state of the upconverting ion Er^3+^ after absorption of two incident 980 nm photons [2,17,18] as illustrated in Figure 4b, inset.

In the analysis of the recorded optical spectra, we observed that the control UCNPs (oleate-capped) showed a bright UC photoluminescence due to a full coverage of the oleate group over their surface which prevents water penetration to quench the UC emission. Unlike the oleate-capped UCNPs, the UC photoluminescence was quenched and increased to retain their good upconversion luminescence efficiency as the silica shell thickness increases from 3 to 8 nm as shown in Figure 4b. This implies that the silica shell coating on UCNPs significantly enhances their upconversion emission efficiency by providing robust physical protection and reducing surface quenching, such as from water molecules. The silica layer acts as a barrier against environmental factors, preserving the UCNPs’ core, while also isolating them from potential quenchers in the surrounding medium. This isolation minimizes nonradiative energy losses at the nanoparticle surface, ensuring higher upconversion efficiency by maintaining the excitation energy within the nanoparticles.

**Figure 5:**
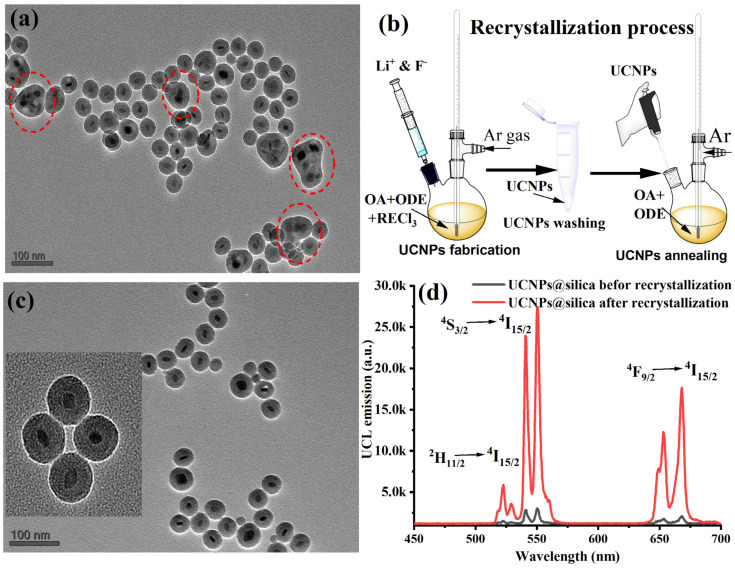
(**a**) Unwanted UCNP aggregates within a silica shell (highlighted in red dashed circles) during a common silica shell formation before the recrystallization process. (**b**) An illustration of the wet chemical annealing procedure used for UCNP recrystallization. (**c**) Post annealing of the synthesized UCNPs in an excess of OA facilitated the successful growth of silica on their surface with single-core UCNPs@silica NPs. (**d**) UC emission recorded from UCNPs@SiO_2_ before and after recrystallization process sensing.

One significant challenge encountered during the growth of silica on upconversion nanoparticles (UCNPs) is the formation of nanoparticle aggregates under a common silica shell. For the nanoparticles to be effectively used in various applications, it is crucial that each UCNP is uniformly and individually coated with silica, with the dispersibility of the nanoparticles being a key factor. In some instances, we noted that UCNPs tended to cluster and form bundles before the application of the silica coating. This led to the production of aggregates with silica coatings as seen in our experiments as shown in Figure 5a. This aggregation is primarily due to the loss of oleic acid (OA) surface ligands during the cleaning process. It has been documented that OA can detach from the nanoparticle surface if UCNPs are excessively treated with ethanol and sonicated, leading to the particles clumping together [29]. To address this issue, Bian et al. developed a method to enhance the crystallinity of α-UCNPs by modifying the crystal edges through a combination of wet chemical treatment and thermal annealing in the presence of OA [29]. We hypothesized that this approach might also enhance the colloidal dispersibility of our UCNPs during silica coating and therefore produce single-core UCNPs@silica NPs.

Toward this goal, we conducted an experiment where UCNPs were annealed at high temperatures with an excess of OA as illustrated in Figure 5b. Experimental details of the UCNPs recrystallization can be found in Section 2. Results of the recrystallized UCNPs were compared with those of nonannealed UCNPs under identical concentrations and conditions during silica shell coating. OA post-treatment markedly improved the colloidal stability of the UCNPs in organic solvents and greatly enhanced the colloidal stability and uniformly of UCNPs. After that, we carried out a silica shell coating around UCNPs with one TEOS concentration (40 µL). Figure 5c shows that post annealing of the synthesized UCNPs in an excess of OA facilitated the successful growth of silica on their surface with single-core UCNPs@silica NPs. Furthermore, we also tested and compared the UC emission of the annealed and nonannealed UCNPs coated with the same thickness silica shell. We found that the UC luminance of the annealed particles enhanced 10× time compared with nonannealed UCNPs at the same silica shell and excitation conditions as demonstrated in Figure 5d.

Finally, in contrast to earlier studies that primarily focused on silica encapsulating of commonly used sodium-based UCNPs without avoiding aggregation of the UCNPs during the silica encapsulation process [30,31,32], our obtained results introduce a refined encapsulation technique for YLiF_4_:Yb,Er UCNPs with a barmaid-like shape with a precisely controlled silica (SiO_2_) shell thickness. In addition, this reported method ensures individual UCNP encapsulation within silica shells, significantly boosting their optical properties, which is critical for their promising application in cutting-edge fields.

As a continuation of this work, we plan to expand on these advancements by developing diamond nanoshells around the synthesized UCNPs@SiO_2_. This initiative aims to mitigate the current issues of background biofluorescence in bioimaging applications where diamond color centers are excited by visible light. Furthermore, we anticipate great potential in exploring Yb-doped YLF UCNPs coated with nanodiamond shells for applications such as laser refrigeration and optical trapping in quantum technologies. These developments could revolutionize the functionality and application of UCNPs in advanced applications.

## 4. Conclusions

We have presented a precise encapsulation of YLiF_4_:Yb,Er upconversion nanocrystals (UCNPs) with silica (SiO_2_) shells using the reverse microemulsion method. The key parameters of this reported method were optimized to produce UCNPs@SiO_2_ core/shell structures with adjustable shell thicknesses and ensure individual UCNP encapsulation. This approach successfully eliminates the occurrence of core-free silica particles and maintains the UCNPs’ inherent upconversion luminescence, critical for their potential applications in many promising applications. Furthermore, our technique effectively prevents the coencapsulation of multiple UCNPs in a single silica shell, a significant achievement that enhances the functionality and applicability of these core/shell structures in advanced technological applications. This work not only contributes significantly to the field of nanomaterial encapsulation but also opens new pathways for utilizing upconversion nanocrystals in innovative ways.

## Figures and Tables

**Table 1:** Parameters for applying a SiO_2_ coating to UCNPs at different concentrations.

Sample	UCNPs (mg)	Igepal CO-520 (mL)	Ammonia (mL)	TEOS (µL)	Silica Shell (nm)	Core-Free Silica (Yes/No)
1	2.8	1.5	0.25	30	6.5	yes
2	4	1.5	0.25	30	6.5	yes
3	5	1.5	0.25	30	6.5	yes
4	6	1.5	0.25	30	6.5	no
5	7	1.5	0.25	30	6.5	no

**Table 2:** Parameters for fabricating UCNPs@SiO_2_ with different TEOS Amounts.

Sample	UCNPs (mg)	Igepal CO-520 (mL)	Ammonia (mL)	TEOS (µL)	Silica Shell (nm)	Core-Free Silica (Yes/No)
6	7	1.5	0.25	10	3.2	no
7	7	1.5	0.25	20	4.5	no
8	7	1.5	0.25	30	6.2	no
9	7	1.5	0.25	40	7.2	no
10	7	1.5	0.25	50	8.1	no

## Data Availability

The data presented in this study are available on request from the corresponding author.
